# Legionella and SARS-CoV-2 Coinfection in a Patient With Pneumonia – An Outbreak in Northern Portugal

**DOI:** 10.7759/cureus.12476

**Published:** 2021-01-04

**Authors:** João Camões, Carolina Tintim Lobato, Francisca Beires, Ernestina Gomes

**Affiliations:** 1 Intensive Care Unit, Unidade Local de Saúde de Matosinhos - Hospital Pedro Hispano, Porto, PRT; 2 Department of Internal Medicine, Unidade Local de Saúde de Matosinhos - Hospital Pedro Hispano, Porto, PRT

**Keywords:** pneumonia, legionella, covid, sars-cov-2

## Abstract

The severe acute respiratory syndrome coronavirus 2 (SARS-CoV-2) pandemic has plagued virtually every continent and country, and Portugal is no exception. The high number of cases has caused a major burden on health services and obvious economic consequences, forcing an important reformulation in the health sectors’ organization. In the past weeks, counties in the country's northern coastal region have reported an increasing number of *Legionella* cases, whose origin is yet to be determined. This exacerbates the already important pressure on the region’s health facilities. We present a case of a patient diagnosed with *Legionella* pneumonia and concomitant coronavirus disease 2019 (COVID-19) pneumonia, highlighting the need for etiological investigation not only for common community agents but also for pandemic pathogens and regional outbreaks.

## Introduction

*Legionella pneumophila* is the bacteria responsible for Legionnaires’ disease, a severe form of pneumonia. Infection by this agent is related to exposure to contaminated water reservoirs, which leads to exporadic community outbreaks. In 2018 a rate of 2.2 cases per 100,000 inhabitants in Europe was notified [1]. Legionnaires’ disease is responsible for 2-11% of community-related pneumonia cases requiring hospitalization [2-4], and 44% of these cases require intensive care admission [5, 6].

The diagnosis of this disease is based on clinical suspicion and laboratory confirmation through a culture of respiratory samples and urine antigen tests. The most frequent clinical manifestations are fever, nonproductive cough, myalgia, and dyspnea. Nevertheless, this infection can also lead to the development of non-respiratory clinical manifestations, such as neurological (confusion and/or headache) and gastrointestinal symptoms (nausea, vomits, and diarrhea). This can pose a diagnostic challenge if these manifestations are not identified early on as signs and symptoms of Legionnaires' disease [7].

In October 2020, a* Legionella* outbreak affected the counties of Vila do Conde, Póvoa do Varzim, and Matosinhos. Portugal's health authorities have detected *Legionella* bacteria in industrial cooling towers nearby. This outbreak has been responsible for at least 72 cases of Legionnaires’ disease, and there have been nine reported deaths to this moment [8]. This outbreak happened during a period of great overload for the Portuguese public health system facing the coronavirus disease 2019 (COVID-19) pandemic, caused by the severe acute respiratory syndrome coronavirus 2 (SARS-CoV-2) virus. COVID-19 pneumonia is also the most severe form of the SARS-CoV-2 virus infection.

The differential diagnosis between these two diseases demands a high level of clinical suspicion and attention, especially during a period of great incidence of SARS-CoV-2 cases. The clinical similarities between these two infections, both causing pneumonia and both presenting with atypical manifestations, make it possible that some cases of Legionnaires' disease were belatedly diagnosed in this context. We report a case of a patient with pneumonia secondary to *Legionella* and SARS-CoV-2, warning for the possibility of concomitant infection, mainly in periods where both are prevalent.

## Case presentation

We present the case of a 53-year-old smoker and obese male, a resident in the Oporto district, who develops a non-productive cough, fever, and small volume hemoptysis at the beginning of November 2020. No other symptoms were recorded. Due to clinical worsening, the patient was referred to the emergency department four days after the beginning of symptomatology.

Initial evaluation showed a tachypneic patient, with peripheral saturation of 90% on room air and with fine crackles in both lung bases upon auscultation. Analytical lab results and gasometric evaluation showed the presence of hypoxemia (partial pressure of oxygen [PO2] 82 mmHg with a fraction of inspired oxygen [FiO2] 28% in Venturi mask, ratio PO2/FiO2 was 292), high inflammatory parameters (C-reactive protein [CPR] 342 mg/dL, procalcitonin 5.2 ng/mL, ferritin 2671 ng/mL) and lymphopenia (500/ uL), without any other major changes.

**Figure 1:**
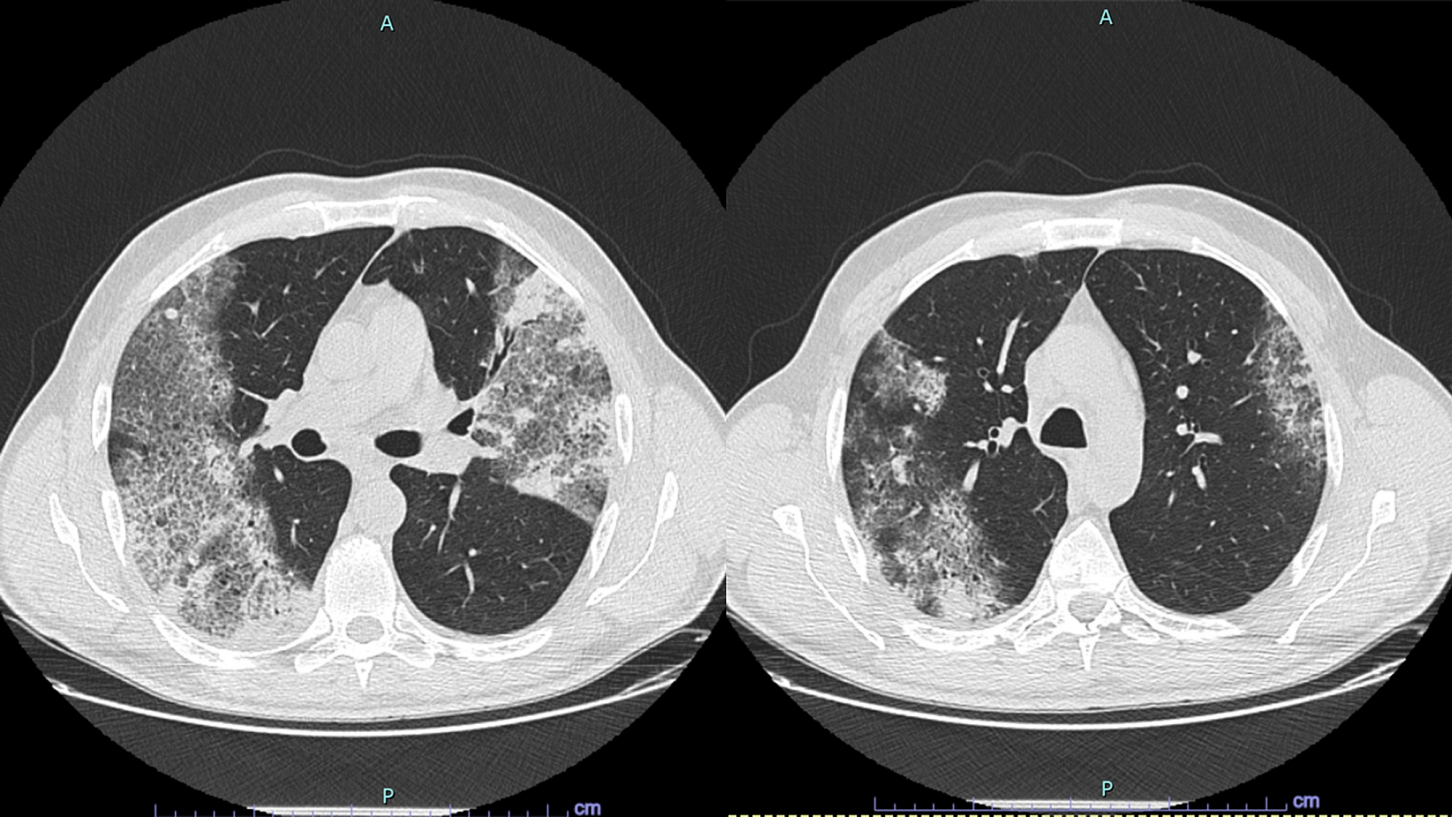
Thoracic CT scan

A thoracic computed tomography (Figure 1) revealed areas of consolidation in the right lower and left upper lobes, coexisting with areas of increased density of stone-like pavement, dispersed in the upper left lobe, as well as in both upper and lower right lobes. The etiological study included two reverse transcription-polymerase chain reaction (rt-PCR) SARS-CoV-2 tests (with a 24-hour difference) that were negative and a* Legionella pneumophila* urinary antigen (immunochromatographic test for detecting *L. pneumophila* serogroup 1), that was positive, although no known positive contacts or site exposure were confirmed. In view of these findings, the patient started levofloxacin 750 mg daily.

In the first hours of hospital admission, the patient showed a worsening condition, with growing respiratory failure (ratio PO2/FiO2 was 230) and polypnea and was admitted to a level 2 (intermediary) unit, where he remained for five days. From an analytical point of view, an initial improvement in the inflammatory parameters reached a plateau phase on the third day of hospitalization (CPR 72 mg/dL). On the fifth day of hospitalization (ninth day of symptoms), marked worsening of respiratory failure was seen ( PO2/FiO2 was 162), as well as increasing polypnea and easy desaturation with minimal physical efforts. Due to this clinical and analytical step back and the high imagiological suspicions, a new rt-PCR SARS-CoV-2 test was performed, which proved to be positive.

The patient was admitted to the intensive care unit (ICU), was intubated and mechanically ventilated for 15 days. He was treated with remdesivir for 10 days, following the latest COVID-19 pneumonia orientations. Administration of dexamethasone was postponed until the eighth day of ICU admission due to the concomitantly active bacterial infection. The patient completed a 21-day course of levofloxacin, accompanied by a favorable clinical evolution with a resolution of the inflammatory syndrome. After 10 days of intubation, a tracheostomy was performed as part of the strategy for mechanical ventilation weaning, which was successfully completed allowing ICU discharge after 20 days.

## Discussion

The clinical presentation of SARS-CoV-2 infection is variable, although the association of fever, dyspnea, and cough are the most common [9]. In this case, due to the high clinical and radiological suspicion of a COVID-19 and bacterial pneumonia, the patient was initially twice-tested to SARS-CoV-2 that were negative. The repetition of tests in negative patients with a suggestive CT scan of SARS-CoV-2 infection has already been advocated by several authors, mainly in those with high clinical and radiological suspicion of COVID-19 pneumonia [10]. In this case, although it is not possible to exclude the nosocomial context for SARS-CoV-2 infection, this was the only case reported among the patient’s hospital contacts, which reinforces the safety policy assumed by health institutions and the low hospital transmissibility when these are met [11]. In our case, it is more likely that the patient was admitted to our institution during the incubation period, with a lower viral load and therefore explaining the negative results of the first two tests. Reviewing the patient’s clinical evolution, we hypothesize that the expression of COVID-19 occurred on the fifth day of hospitalization when a clinical deterioration was seen. Dexamethasone and remdesivir were started in accordance with the state of the art in the present time [12, 13].

In the *Legionella* outbreak in northern Portugal, the official source is yet to be determined. The possibility of *Legionella *outbreaks during periods of population confinement has already been addressed by an Italian group, in which the temporary and/or permanent closure of public establishments may promote the growth of these species in water reservoirs and, in the re-opening setting, may cause local or regional outbreaks [14]. While this specific outbreak's origin remains under investigation, every day, more cases are reported, and hospitalizations are rising; this increases the already high demand for hospital beds in the northern area of the country.

## Conclusions

The authors warn of the possibility of concomitant respiratory infection by SARS-CoV-2 and *Legionella*. The latter should be considered in the presence of a clinical and epidemiological context, mostly in the northern region of Portugal.
